# To Wean or Not to Wean: The Role of Autologous Reconstructive Surgery in the Natural History of Pediatric Short Bowel Syndrome on Behalf of Italian Society for Gastroenterology, Hepatology and Nutrition (SIGENP)

**DOI:** 10.3390/nu12072136

**Published:** 2020-07-18

**Authors:** Teresa Capriati, Antonella Mosca, Tommaso Alterio, Maria Immacolata Spagnuolo, Paolo Gandullia, Antonella Lezo, Paolo Lionetti, Lorenzo D’Antiga, Fabio Fusaro, Antonella Diamanti

**Affiliations:** 1 Artificial Nutrition Unit, Bambino Gesù Children Hospital, 00165 Rome, Italy; teresa.capriati@opbg.net; 2 Hepatology Unit, Bambino Gesù Children Hospital, 00165 Rome, Italy; antonella.mosca@opbg.net (A.M.); tommaso.alterio@opbg.net (T.A.); 3 Department of Translational Medical Science, Section of Pediatrics, University of Naples Federico II, 80138 Naples, Italy; mispagnu@unina.it; 4 Gastroenterology Unit, G.Gaslini Institute for Maternal and Child Health, IRCCS, 16145 Genova, Italy; paologandullia@gaslini.org; 5 Division of Nutrition, Regina Margherita Children’s Hospital, 10126 Turin, Italy; alezodott@gmail.com; 6 Department of Neuroscience, Psychology, Pharmacology and Child’s Health, University of Florence, Meyer Hospital, 50139 Florence, Italy; paolo.lionetti@unifi.it; 7 Paediatric, Hepatology, Gastroenterology and Transplantation, Hospital Papa Giovanni XXIII, 24127 Bergamo, Italy; ldantiga@asst-pg23.it; 8 Department of Medical and Surgical Neonatology, Bambino Gesù Children’s Hospital, 00165 Rome, Italy; fabio.fusaro@opbg.net

**Keywords:** parenteral nutrition, autologous gastrointestinal reconstructive surgery, short bowel syndrome, intestinal failure, liver disease

## Abstract

Pediatric Short Bowel Syndrome (SBS) can require prolonged parenteral nutrition (PN). Over the years, SBS management has been implemented by autologous gastrointestinal reconstructive surgery (AGIR). The primary objective of the present review was to assess the effect of AGIR on weaning off PN. We also evaluated how AGIR impacts survival, the need for transplantation (Tx) and the development of liver disease (LD). We conducted a systematic literature search to identify studies published from January 1999 to the present and 947 patients were identified. PN alone was weakly associated with higher probability of weaning from PN (OR = 1.1, *p* = 0.03) and of surviving (OR = 1.05, *p* = 0.01). Adjusting for age, the probability of weaning off PN but of not surviving remained significantly associated with PN alone (OR = 1.08, *p* = 0.03). Finally, adjusting for age and primary diagnosis (gastroschisis), any association was lost. The prevalence of TX and LD did not differ by groups. In conclusion, in view of the low benefit in terms of intestinal adaptation and of the not negligible rate of complications (20%), a careful selection of candidates for AGIR should be required. Bowel dilation associated with failure of advancing EN and poor growth, should be criteria to refer for AGIR.

## 1. Introduction

Short bowel syndrome (SBS) is the most common cause of intestinal failure (IF) in children [1]. It results from the extensive resection of the small intestine due to congenital malformations or post-natal acquired diseases [2]. Intestinal atresias, midgut volvulus, total aganglionosis, necrotizing enterocolitis (NEC), mesenteric vascular insufficiency, cancer and abdominal trauma are the main causes of pediatric SBS [1,2,3]. Extensive bowel resections lead to impaired nutrients, water and electrolyte absorption, thus leading to dependency on parenteral nutrition (PN) for non-predictable periods until “intestinal adaptation” occurs [2,3]. Intestinal adaptation is the overall structural and functional changes of the remaining gut and it begins shortly after resection [4,5,6,7,8,9]. It is based on the development of compensatory mechanisms that improve the absorptive capacity of the residual bowel after the resection, consisting in: (1) increased intestinal mass and surface area; (2) enterocyte and crypt cell proliferation; (3) improved villi height and crypts depth;(4) development of hyperplasia and hypertrophy of the smooth muscle layers. Adaptation begins 24–48 h after surgical resection and it advances during the initial 4–24 months following resections [5,6]. Overall, these changes result in prolonged intestinal transit time and improved absorption. Furthermore, after intestinal resection, the motility pattern of residual gut also seems to be abnormal [5], due to the shorter duration of migrating motor complex cycle [6]. Several factors may impact the trend of bowel adaptation, including gastrointestinal regulatory peptides, growth factors, hormones and cytokines [4,5,6,7,8,9]. In some patients, the above reported pathophysiological changes can result in bowel dilation and subsequent dysmotility and stasis [10], which promote small bowel bacterial overgrowth (SBBO) [11]. SBBO can cause malabsorption, bacterial translocation with systemic infection, eventually contributing to the development of liver disease (LD) [11,12]. LD, in turn, negatively impacts ongoing adaptation and the ability to wean off PN [12,13].

Nowadays more SBS infants are able to survive, but long-term morbidity requires a comprehensive, multidisciplinary approach and usually a long-term use of PN [14,15,16,17,18,19,20,21]. Multidisciplinary management has a key role in promoting intestinal autonomy and in avoiding long-term PN complications. The multidisciplinary approach should provide integrated care of inpatients and outpatients by favoring the coordination of surgical, medical, and nutritional management [14,15,16,17,18,19,20,21]. Nutritional care generally includes the following steps: (1) early managing of fluids and electrolyte losses before starting PN and enteral nutrition (EN); (2) providing adequate PN, for growth and normal development; (3) promoting intestinal rehabilitation by optimizing EN;(4) discharging the patients with predicted long-term PN on home parenteral nutrition (HPN) [22].PN is essential to sustain malabsorption, but it should be modulated, according to the amount of tolerated enteral intake, and it should be reduced as soon as the intestinal tolerance improves. EN should be started when the post-operative ileus resolves and progressively increases according to intestinal tolerance. Oral nutrition (ON) should be promoted to reach a normal feeding behavior and it is the preferred feeding way in patients needing HPN programs [22].

Surgery, by the so called “autologous gastrointestinal reconstruction” (AGIR) of the remaining bowel, is another relevant part of the work of a multidisciplinary team [23]. AGIR facilitates the adaptation process, because it increases the length of the small intestine and the absorptive mucosal surface [24]. AGIR reduces segmental bowel dilation, thus preventing SBBO [24]. Longitudinal intestinal lengthening and tailoring (LILT) was the first to be approached among the AGIR procedures and it was first described by Adrian Bianchi [25]. In the LILT procedure, an avascular space is created longitudinally along the mesenteric border of a dilated loop of bowel [25]. The bowel is then split lengthwise, taking care to allocate alternating blood vessels to each side [25]. Each side of the split bowel is then tubularized, generating two “hemi-loops” that are anastomosed [25]. After LILT, a loop of bowel that is twice the length of the original and half the original diameter is created. Another procedure that often is proposed is the serial transverse enteroplasty (STEP) [26]. In the STEP procedure, the intestinal lumen is narrowed by firing a series of staples perpendicularly to the long axis of the bowel in a zig-zag pattern without interfering with the blood supply of the bowel. These techniques have been applied to intestinal segments with dilation >4 cm [27]. A new technique, that can also be planned in patients with a lesser degree of bowel dilatation (<4 cm) is the spiral intestinal lengthening and tailoring (SILT) [28]. SILT has the advantage of minimal handling of the mesentery and of not severely altering the orientation of the muscle fibers asSTEP procedures [28].

Moreover, in some patients, procedures to slow intestinal transit, such as colonic interposition and reversed segments, might be required alone or in combination with the above lengthening procedures [29]. In cases of extreme short bowel, a colonic segment can be interposed between the remaining small bowel, either in iso-peristaltic or anti-peristaltic fashion [26,29]. The colon undergoes fewer peristaltic cycles as compared to small bowel and contributes to the prolonged intestinal transit time observed with the procedure, whilst an anti-peristaltic method of application further reduces the rate of transit by causing a reversed propulsion effect [29]. A reversed segment, or anti-peristaltic segment, is a procedure implemented to slow transit time, allowing for improved nutrient absorption [29]. Based on advances in the management of SBS, it is now known that approximately 70% of all SBS patients will adapt and become independent from PN [30].

In SBS children, gut has a great potential for spontaneous growth; therefore, the key question is: ”Should we wait for natural adaptation or interfere with the natural course by AGIR?” Thus, the main purpose of the present review was to assess the effect of AGIR on weaning off PN in children with SBS. Furthermore, we detected the impact of AGIR on survival, on trend towards irreversible forms of Intestinal Failure (IF) requiring transplantation (Tx) and on the development of liver disease (LD).

## 2. Methods

### 2.1. Literature Search Strategy

According to the PRISMA (Preferred Reporting Items for Systematic Reviews and Meta-analyses) statement [31], we conducted a systematic literature search to identify relevant studies published on the PubMed database from January 1999 to the present. Researched items were: ”spiral intestinal lengthening and tailoring AND short bowel syndrome”; ”spiral intestinal lengthening and tailoring AND enteral nutrition”; ”spiral intestinal lengthening and tailoring AND parenteral nutrition”; ”serial transverse enteroplasty AND short bowel syndrome”; “serial transverse enteroplasty AND enteral nutrition”; “serial transverse enteroplasty AND parenteral nutrition”; “longitudinal intestinal lengthening and tailoring AND short bowel syndrome”; ”longitudinal intestinal lengthening and tailoring AND enteral nutrition”; ”longitudinal intestinal lengthening and tailoring AND parenteral nutrition”; ”parenteral nutrition AND short bowel syndrome”. The results were filtered according to: (a) Age: 0–18 years; (b) Species: Humans; (c) Language: English.

### 2.2. Eligibility Criteria and Study Selection

Inclusion criteria were the following:

1. SBS acquired at neonatal age;
2. SBS patients PN-dependent at inclusion;
3. Follow-up length: 1–5 years;
4. Follow up beginning before 2000;
5. Lack of reference to the weaning off PN trend.

Exclusion criteria were the following:

1. Single cases or case series with less than three patients;
2. Patients treated with known intestinal growth factors, such as Glucagon-like peptide 2, Glucagon-like peptide 1, insulin, glutamine and Growth hormone;
3. Secondary literature (review, editorial, position paper, guidelines).

Two authors separately screened the studies for eligibility based on inclusion/exclusion criteria. Articles were screened in two stages. First, titles and abstracts were reviewed to identify potentially relevant articles. Full-text of those abstracts which met the inclusion criteria was retrieved and independently reviewed in the second stage of the assessment.

The papers included [21,30,32,33,34,35,36,37,38,39,40,41,42,43,44,45,46,47,48,49,50,51,52,53,54,55,56,57,58,59,60,61,62] were subdivided into two groups according to the treatment. Therefore, references [21,30,32,33,34,35,36,37,38,39,40,41,42,43] included patients treated with PN alone or series in which less than 10% of patients were treated with combined PN and AGIR (Group 1). References [44,45,46,47,48,49,50,51,52,53,54,55,56,57,58,59,60,61,62] reported the trend of patients all treated with combined PN and AGIR (Group 2).

- The primary outcome was the prevalence of weaning off PN, compared between Groups 1 and 2 and between a subset of patients in Groups 1 and 2, aged <24 months and ≥24 months
- Secondary outcomes were survival, Tx and LD.

### 2.3. Data Extraction, Synthesis and Analysis

Data obtained from the selected articles were gathered and entered into tables. Data included the following information: author; year, and country of publication; follow up length; number of patients; age and gender; age at AGIR; type of AGIR; etiology of SBS; length of residual small bowel (SBL) before and after AGIR surgery; presence of ileocecal valve (ICV); presence of >50% colon; prevalence of PN weaning, survival, TX and LD disease.

Statistical analyses were performed with MEDCALC (version 19.0.7, Ostend, Belgium). Normally distributed data are described as means ±standard deviations (SD) and data not normally distributed are expressed as median and IQR. Categorical variables were summarized as percentages and compared by χ^2^ test or χ^2^ test for trend, as appropriate.

Differences between groups were tested by Kruskal–Wallis test for not normally distributed data; *t*-student or U-test were used for normally distributed data. Correlations between PN/AGIR and outcomes were assessed by Spearman’s test.

Based on the results of univariate analyses, a binary logistic regression (ORs, 95%CIs) was built (PN = 0, AGIR = 1). A binary model was used to predict PN (=0) as exposure with respect to PN + AGIR (=1) to test whether PN versus AGIR is independently associated with main outcome (weaning off PN) and secondary outcomes (survival, need for Tx and development of LD). Then the model was adjusted for the variables significantly different by group at the univariate analysis. Results were considered significant for *p* < 0.05.

## 3. Results

**Figure 1:**
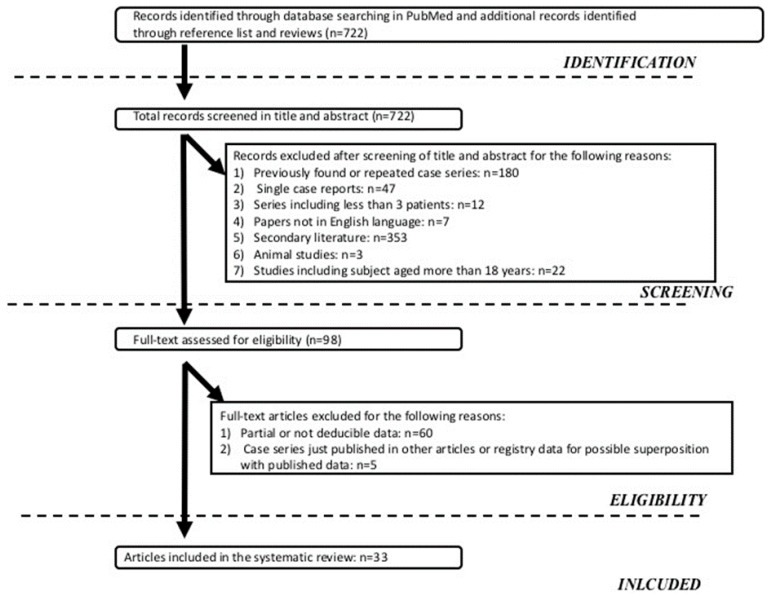
Algorithm of studies selection.

The systematic literature search identified 722 potentially relevant articles that matched the search criteria, as shown in Figure 1. After considering our inclusion and exclusion criteria, 33 articles (PN + AGIR, *n* = 19, and PN alone, *n* = 14) were selected.

### 3.1. Summary of the Series Including Patients Treated with PN + AGIR

Overall intestinal adaptation ranged from 6% [46] to 87% [56] at the final evaluation. STEP and LILT procedures elongated the bowel up to 75% and 100%, respectively, both requiring adequate bowel dilation and fresh and scar-free mesentery to allow for extensive intestinal handling [44,45,46,47,48,49,50,51,52,53,54,55,56,57,58,59,60,61,62].

The chance to be weaned off PN following STEP and LILT procedures ranged from6% [46] to 67% [59] and from 55.5% [45] to 100% [56], respectively. Only 11 studies assessed the development of LD that ranged from 2.7% [44] to 67% [59]; however, LD was considered as an indication of AGIR in many studies and not as a complication. Furthermore, 4.5% [45] to 33% [59] underwent transplantation.

Only a few patients, in the report of Coletta and coworkers, were submitted to SILT, and all resulted in being weaned off PN following a 26-month follow up [48]. In this study nevertheless, SILT was not the primary surgery in all patients and it was combined with STEP [48]. All patients following SILT underwent progressively normalized liver function tests [48].

Interestingly, a few studies reported the results of rarer AGIR approaches, such as colonic interposition [47], reversed segment [47], duodenal lengthening or enteroplasty in dilated duodenum [51,56], often performed in combination with STEP or LILT. These techniques could give benefits in neonates [55,61] and in patients with ultrashort bowel syndrome [55].

Patients not successfully treated with the first STEP were often submitted to second or even third procedure (ReSTEP) [49,50,57]. We found that neither demographic data nor the etiology of SBS were predictive of need for ReSTEP. The chance to be weaned from PN was 18% one year, following the first STEP, and 0% following the ReSTEP and, as a consequence, the caloric intake by EN increased by26.0% following first STEP and only by4.7%following ReSTEP. Similarly, bowel length improved more significantly after STEP (51%) than ReSTEP (20%) [49]. The lack of an ileocecal valve was the only factor predicting the need for ReSTEP [50].

The probability to achieve enteral autonomy decreased in re-dilated patients following STEP [57].

Mucosal inflammation seemed to be correlated to persisting bowel dysfunction and the need for ReSTEP, especially in the absence of the ileocecal valve [46].

### 3.2. Summary of the Series Including Patients Treated with PN Alone

SBS is the main indicator of long-term PN programs, as shown by three consecutive case series that focused on HPN [33,37,39] in which SBS was 49%, 59% and 65% of the indications of HPN programs.

With regard to the outcomes of SBS, especially the reaching of enteral autonomy, most authors agree on the low impact of the of primary disease of SBS [30,38] and on the great relevance ofresidual bowel anatomy [30,32,34,37,39,40].

Belza et al. [31] reported that enteral autonomy was achieved in 70% of the patients. In this series, the subjects who remained on HPN had significantly shorter residual small bowel (29 vs. 69 cm *p* < 0.0001) and colon length (65 vs. 86 cm *p* < 0.001).In this series 80% to 100% of patients with expected gestational age residual bowel length >50% achieved enteral autonomy irrespective of the amount of residual colon within 1–2 years. In infants with expected gestational age residual bowel length <50%, the colon played a crucial role in weaning instead of PN. Lakananurak et al. [34] reported that, over19SBS children,51% were dependent on PN, following a median follow up of 48 months. In this experience, residual colon ≥50% was significantly associated with survival and enteral autonomy, especially in patients with very short bowel syndrome. Capriati et al. [32] found that, in SBS patients with a residual bowel length ≤40 cm, 51% were weaned from PN 48 months following the last resection. In this series the probability of weaning off PN was 87% to 100% in patients with remaining bowel length >20 cm. Among those with residual bowel length <20 cm, only 25% achieved intestinal autonomy; however, in this subgroup the chance to be weaned from PN was 54% and 57% if ICV and ileum, respectively have been maintained.

Another concern with regard to the attitude of SBS children to be weaned from PN is bowel dilation. Hukkinnen et al. [36] founded that small bowel dilation is associated with prolonged dependency on PN and impaired survival. Chester et al. [38] followed 29 SBS patients between 2000 and 2015 and, according to the report from Hukkinen and co-workers, they founded that short bowel dilation predicted failure to achieve enteral autonomy. Furthermore, the length of the residual bowel and primary diagnosis (atresias) predicted the development of bowel dilation.

Some reports focused on the potential markers for monitoring small bowel adaptation, which has been identified with citrulline in many cases. Chiba et al. [35] found that serum citrulline is a predictor of PN independence, while fasting serum gastrin and GLP-2 are predictors of intestinal adaptation. Bailly-Bouta et al. [42] showed that plasma citrulline had positive correlation with residual short bowel length. In this series it increased over time during or after weaning from PN but remained stable or low in patients who continued to need PN. Additionally, they showed that patients with residual bowel length <40 cm and plasma citrulline concentration <11 µmol/L remained dependent on PN at the final evaluation.

Some surveys evaluated the impact on the era on SBS outcomes [21,32,38,39,40] and all found that survival and LD greatly increased over the years. However, the probability of developing enteral autonomy remained <75% in the majority of the surveys and appeared dependent on the residual anatomy of bowel length.

### 3.3. Summary of the Patients Characteristics

The review included 947 patients (PN + AGIR, *n* = 320, and PN alone, *n* = 627), as shown in Table 1 and Table 2. The overall management of AGIR is detailed in Table 3. Complications following AGIR occurred in 20% of the patients. Among the patients treated with PN + AGIR, we could extrapolate several subjects who had undergone exclusively STEP (*n* = 242), as shown in Table 4. Regarding the distribution by age, we found that 214 and 284 patients aged less than 24 months and 198 and 106 aged 24 months or older, were treated with PN alone and PN + AGIR, respectively, as shown in Table 5. The definition of LD was very variable; therefore, taking into account the definition employed in each report and in order to make the LD classification homogeneous, we re-defined it as abnormal liver function tests (LFTs) (plasma aminotransferase and gamma-glutamyl transferase) or as intestinal failure-associated liver disease (IFALD), if abnormal LFTs were combined with hyperbilirubinaemia.

#### 3.3.1. Univariate Analysis

Patients had significantly different distribution for age and prevalence of gastroschisis as a primary disease, and residual colon >50%, if compared to those PN treated with PN + AGIR and PN + STEP. Regarding the outcomes, the major difference was that the prevalence of weaning of PN and LD were both significantly higher in the group treated with PN alone than in the groups receiving PN+any surgery, as shown in Table 4.Univariate analysis, according to age (<24 months and ≥24 months), found that NEC, as a primary diagnosis, was differently distributed in patients treated with PN alone. With regard to the outcomes, the prevalence of weaning from PN and survival was significant between the two groups treated with PN + AGIR and PN alone, respectively, as shown in Table 5.

#### 3.3.2. Logistic Binary for PN Alone/AGIR and PN Alone/STEP

Finally, we investigated the associations between PN and outcomes. Based on univariate analysis, we found that the differences in the distribution of primary diagnosis (gastroschisis) and in age (<24 and ≥24 months), could represent the variables able to influence the impact of PN alone on the outcomes over PN + AGIR. Therefore, we built a binary logistics analysis of three. We found that PN alone, if compared to PN + AGIR, was significantly associated with higher probability to wean from PN and to survive. Adjusting for age, the probability to wean off PN but to not survive remained significantly associated with PN alone. Finally, adjusting for age and primary diagnosis of gastroschisis, any association was lost. When we considered only STEP procedures, the results were similar to overall AGIR, resulting in PN alone associated with better chance to wean from PN in the model adjusted for age but not in the model adjusted for age and primary diagnosis of gastroschisis. In this analysis, we found, nevertheless, no association between PN alone and higher probability to survive, as shown in Table 6.

## 4. Discussion

The progress of the multidisciplinary management has greatly improved the outcomes of SBS over the years. Nowadays, more than >90% of SBS patients survive, as assessed in the present review, and, therefore, careful modulation by a multidiscipline team is mandatory to improve the outcomes. The key objectives of any multidisciplinary intestinal rehabilitation program are to promote gut adaptation while minimizing the progression of complications related to prolonged PN exposure. The focus of the present review was to evaluate the specific impact of AGIR, as part of a comprehensive multidisciplinary program, on the natural history of pediatric SBS and especially on the probability of developing enteral autonomy.

We found that AGIR does not add advantages on the natural course of SBS in terms of acquiring enteral autonomy. In particular, if we compared first and last published surveys in patients treated with PN alone, we found that, in 2008, Salvia and coworkers reported a rate of enteral autonomy of 52% over a median follow up of 34.5 months and, in 2019, Belza and coworkers reported a rate of weaning off PN of 70% [43] over a median follow up of 30 months. In patients treated with PN + AGIR, Avery Ching and coworkers in 2009 reported a rate of weaning of 38% over a median follow up of 23 months [60]. In 2019, Fitzgerald et al. reported a rate of enteral autonomy of 42% over a median of 47 months [44]. Furthermore, age at surgery failed to demonstrating any advantage in terms of acquiring enteral autonomy. Interestingly, the natural course of SBS, due to gastroschisis, was particularly disadvantaged by the surgery, because it had been burdened by highest mortality rate and long-term dependency on PN. It has been suggested that gastroschisis may be unfavored in terms of adaptation by the underlying severe dysmotility [63]. In animal models, gastroschisis is associated with hypertrophy and hyperplasia of both the circular and longitudinal muscle layers [64,65] and to a late differentiation of the intestinal cells of Cajal [66], which can all explain the dysmotility. Gastroschisis accounted for one third of the indications of AGIR and STEP and that suggests that such patients may be more prone to develop dilation as a consequence of dysmotility, than other primary diagnoses of SBS. STEP, nevertheless, alters the muscle fiber orientation and therefore can worsen the distinctive dysmotility of gastroschisis [66]. In the present review, the overall probability to achieve intestinal autonomy was <50% after AGIR. This finding is similar to that reported by Jones et al., who published the largest report on the STEP, including 111 patients from the International STEP Registry [67]. The International STEP Data Registry, created in 2004, collected information on patients undergoing the STEP procedure from September 2004 to January 2010. Fourteen of the 111 patients who were included in the Registry were excluded due to inadequate follow-up. Of the remaining 97 patients, 11 patients died (11%), and five progressed to intestinal transplantation (5%). Of the 78 patients who were ≥7 days of age and required parenteral nutrition (PN), at the time of STEP, 37 (47%) achieved enteral autonomy after the first STEP [67]. Therefore, the STEP outcomes found in the present review were very similar to those reported by the STEP Registry.

A great concern of any surgery is the probability of complications, which was 20% in the reports selected for this review. The most frequent complications are stricture/obstruction [44,47,52,53,55,58,59,60], infections [44,47,48,53,59], abdominal adhesions [44,47,54,60] and entero-cutaneous fistula [47,52]. In view of the poor results in terms of intestinal adaptation, if compared to the natural course and the non-negligible rate of complications, the surgical approach should be reserved for selected candidates. In particular, the coexistence of intestinal dilation and signs and symptoms of SBBO with inability to advance EN and/or poor growth, should be the true indication of AGIR. Bowel dilatation is a natural consequence of adaptation [6,7,8,9]; therefore, surgery should be delayed if dilation does not cause SBBO and related symptoms [52,53].

A relevant issue in the management of children with SBS is the potential development of LD. IF-related and PN-related factors play both a key role in the development of LD. Lack of enteral feeding, disturbed entero-hepatic bile flow, presence of inflammation, oxidative stress, immaturity of the liver and infections are the main IF-related factors. Imbalance (deficiency/excess) of parenteral nutrients, such as excess of energy, excess or type of amino-acids solutions and type of lipid emulsion may impact the development of LD [67]. The present review found that the probability of developing LD was significantly higher in patients treated with PN alone that in those treated with PN + AGIR. This difference decreased if we considered only IFALD and not the wall constellation of LD. The definition of LD in this review is affected by two problems: (a) the definition of LD has changed over the years and, therefore, data regarding the liver involvement are difficult to compare between the various studies; (b) many surgical reports have considered LD as an indication of surgery rather than a complication [45,49,61]. Although a clear definition of LD could be a concern in this review, AGIR certainly plays a key role in preventing LD by improving intestinal dilation and SBBO [68]. Therefore, the development of early signs of liver involvement, if an intestinal dilation is present, could be included among the selection criteria for AGIR.

As a last aspect, we observed that the proportion of patients who were referred to transplantation procedures was negligible and, overall, less than 10%, but higher in patients undergoing PN + AGIR. In patients undergone PN alone it was around 5%. These data are in line with the decline in indications for bowel transplantation in recent years [69].

One of the most important limitations of this review was the lack of comparison between STEP and LILT outcomes because our eligibility criteria precluded the inclusion of reports regarding LILT alone. However, in the literature, the probability of weaning off PN was reported as not different between LILT and STEP [70,71,72]. A further potential limitation of the review could be the inclusion of several centers with different clinical experiences and practices that could have a different impact on the results.

## 5. Conclusions

In conclusion, in view of the low benefit in terms of intestinal adaptation and the not negligible rate of complications, AGIR should not be proposed to all patients with SBS but the best candidates should be carefully selected. The requirements to refer for AGIR should be radiologically evident bowel dilation associated with signs or symptoms of SBBO. Among the clinical evidences for SBBO failure of advancing EN and poor growth should be emphasized. The presence of early signs of IFALD should also be considered in the presence of intestinal dilation as a factor in favor of eligibility for AGIR. These conclusions are mostly based on retrospective studies and with a sometimes very limited number of patients. The future availability of prospective studies that include a larger number of patients may help to better define AGIR’s role in the intestinal rehabilitation of pediatric SBS.

## Figures and Tables

**Table 1:** Overview of the studies including patients treated with PN alone.

Author(Year)	Country	Study-Period	Study Population *	Number of Patients Weaned from PN
Belza (2019)	Canada	2006–2013	120	84
Capriati (2018)	Italy	2008–2016	19	7
Brown (2018)	UK	2001–2016	15	4
Lakananurak (2018)	Thailand	2005–2015	19	9
Chiba (2017)	Japan	2000–2015	7	5
Hukkinen (2017)	Finland	2002–2015	61	49
AbiNader (2016)	France	2000–2013	148	91
Chester Ives (2016)	USA	2000–2015	29	16
Petit (2016)	France	2000–2009	98	57
Schurink (2014)	Netherlands	2001–2009	19	16
Nusinovich (2012)	USA	2000–2010	20	11
Sigalet (2009)	Canada	2006–2009	22	18
Bailly-Bouta (2008)	France	2001–2004	31	9
Salvia (2008)	Italy	2003–2004	19	10

PN: Parenteral nutrition; * in this sample 54 patients underwent combined parenteral nutrition + autologous reconstructive gastro-intestinal surgery.

**Table 2:** Overview of the studies including patients treated with PN + AGIR.

Author (Year)	Country	Period of Study	Study Population	Number of Patients Weaning of PN
Fitzgerald (2019)	Canada	2003–2016	36	15
Shah (2019)	USA	2004–2014	22	11
Mutanen (2019)	Finland	2003–2014	15	1
Pederiva (2018)	UK	2002–2012	43	25
Coletta (2017)	UK	2012–2017	5	0
Barrett 2017)	USA	2003–2014	17	6
Wester (2016)	Finland	2004–2015	27	16
Bueno (2015)	Spain	2005–2013	3	1
Oh (2014)	USA	2004–2011	15	5
Mercer (2014)	USA	2006–2011	51	24
Javid (2013)	USA	2004–2011	16	9
Wales (2013)	Canada	2009–2011	5	2
Almond (2013)	UK	2004–2011	8	7
Kang (2012)	USA	2002–2011	16	8
Leung (2012)	Hong Kong	2007–2010	4	1
Lourenco (2012)	Portugal	2006–2008	3	2
Ching (2009)	USA	2002–2008	16	6
Wales (2007)	Canada	2003–2006	14	7
Duggan (2006)	USA	2002–2014	4	2

PN: Parenteral nutrition; AGIR: autologous reconstructive gastro-intestinal surgery.

**Table 3:** Indications, types of surgery and complications [Ref. n°].

INDICATIONS
*Nutritional* [52,60,62] Failure to progress EN
*Digestive* [45,49,60,61] At least 10 cm of small bowel+>50% of the colon, dysmotility, vomiting, bacterial overgrowth, obstructive symptoms, increased secretions, IFALD
*Extra-digestive* [49] Fluid and electrolyte problems, D-lactic acidosis, vascular access problems
*Biochemistry/Imaging based* [45] Small bowel > 35 cm + increased bilirubin level + normal INR; small bowel ≥50 cm of bowel+portal hypertension+ hypersplenism ± thrombocytopenia + normal INR
*Imaging based* [48,52,58,61] Gut dilatation, as shown by upper gastrointestinal contrast study with diameter >3.5 cm or >2 vertebral bodies
**TYPE OF SURGERY**
STEP [44,45,46,47,49,50,51,52,53,54,55,56,57,58,59,60,61,62], LILT [44,47,57], SILT [48], reverse segment [47], colonic interposition [47], tapering enteroplasty [47,51,56]
**COMPLICATIONS (in 64/320 Patients)**
Staple leak [44,61], stricture/obstruction [44,47,52,53,55,58,59,60], bleeding, abdominal adhesions [44,47,54,60], entero-cutaneous fistula [47,52], bowel wall hematoma [52], infections [44,47,48,53,59], intra-abdominal abscess [55], ulcers [54,61], D-lactic acidosis and abdominal distension [48,49,50,51,52,53,54,55,56,57,58,59]

EN: Enteral Nutrition; IFALD: Intestinal failure-associated liver disease; INR: normal international normalized ratio; STEP: serial transverse enteroplasty; LILT: longitudinal intestinal lengthening and tailoring; SILT: spiral intestinal lengthening and tailoring.

**Table 4:** Summary of the results according to treatments.

	PN	PN + AGIR	PN + STEP	*p* PN vs. Surgery	*p* PN vs. STEP
**General Data**		
	N° Pts	627	320	242	0.09	0.04
	N° studies	13	19	15	/	/
	Male/Female	242/176	155/126	120/91	0.06	0.06
Follow up (mo)	53.3 ± 41.3	36.2 ± 15.9	28.4 ± 11.8	0.06	0.03
GA	34.3 ± 0.8	34.5 ± 0.9	34.1 ± 3.8	0.66	0.87
<24 mo/≥24 mo	59/41	67/33	81/19	0.001	0.0001
**Etiology (%)**					
NEC	29.5	15	15	0.06	0.12
Gastroschisis	17	32.5	33	0.04	0.02
Volvulus	16.7	11.8	9	0.64	0.55
Multiple atresias	17.3	24.6	26	0.29	0.24
Hirshprung	6.2	2.5	2.4	0.66	0.56
Combined	1.6	6.8	6.6	0.18	0.27
Other	3.9	0.3	0.4	0.04	0.04
**Residual Bowel Anatomy (%)**					
Length BS	45.7 ± 20.9	50.8 ± 22.6	57.2 ± 22.0	0.74	0.68
Length AS	NA	80.3 ± 33.5	90.3 ± 32.5	UV	UV
ICV+	48.5	16.5	26	0.003	0.10
Colon>50%	43.8	62.5	96.2	0.007	0.001
Age at surgery		39.4 ± 56.6	22.7 ± 21.3	UV	UV
**Outcomes (%)**					
Weaning off PN	61.6	46.2	43	0.03	0.01
Survival	91.5	95	94.2	0.72	0.82
Tx	5.4	7.5	9.5	0.24	0.045
LD	30.4	12	14.3	0.001	0.001
IFALD	23.2	12	14.3	0.01	0.06
Abnormal LFTs	33/461 (7.1)	NA	NA	UV	UV

PN: Parenteral nutrition; AGIR: autologous reconstructive gastro-intestinal surgery; STEP: serial transverse enteroplasty; N°: number; GA: gestational age; NEC: necrotizing enterocolitis; BS: before surgery; AS: after surgery; ICV+: ileocecal valve present; UV. Unvaluable; Tx: transplantation; LD: liver disease; IFALD: intestinal failure-associated liver disease; LFTs: abnormal liver function tests; NA: not available.

**Table 5:** Summary of the results according to age.

	<24 Months		≥24 Months		*p* NP	*p* Surgery
	PN	AGIR	NP	AGIR		
**General Data**						
N° Pts	284	214	198	106	/	/
N° studies	8	12	3	7	/	/
M/F	56/44	55/45	48/52	55/45	0.74	0.38
Follow up	41.7 ± 27.4	32.1 ± 12.2	59.7 ± 23.1	36.9 ± 13.2	0.06	0.09
Age at surgery		13.6 ± 4.3		43.8 ± 28.7		0.02
**Etiology (%)**						
NEC	50	15	22.7	14	0.004	0.42
Gastroschisis	20	38	19.1	22	0.84	0.23
Volvulus	23.1	8	23.7	19	0.85	0.90
Atresias	20.4	27	34 (17.1)	22 (21)	0.95	0.79
Hirshprung	33/284 (11.6)	6 (3)	25 (12.6)	2 (2)	0.77	0.15
Combined etiologies	25/186 (13.4)	16 (7)	0 (0)	6 (6)	/	0.38
Other	14/186 (7.5)	1 (0.4)	9 (4.5)	0 (0)	0.55	/
**Residual Bowel Anatomy**						
Length BS	51.6 ± 18.6	55.8 ± 17.6	51.8 ± 2.5	45.2 ± 29.9	0.87	0.45
Length AS		84.5 ± 33.3		76.9 ± 36.9	/	0.37
ICV+	54.8	30	47.9	24	0.36	0.96
**Outcomes**						
Weaning off PN	59.5	44	55.5	84	0.59	0.01
Survival	79.9	95	98.5	94	0.03	0.79
Tx	7.2	9	5.9	4	0.48	0.50
LD	1	6	17.3	19	0.35	0.75

PN: Parenteral nutrition; AGIR: autologous reconstructive gastro-intestinal surgery; STEP: serial transverse enteroplasty; GA: gestational age; NEC: necrotizing enterocolitis; BS: before surgery; AS: after surgery; ICV: ileocecal valve; Tx: Transplantation; IFALD: intestinal failure-associated liver disease; LFTs: abnormal liver function tests.

**Table 6:** Logistic binary PN vs. AGIR and PN vs. STEP.

	PN vs. AGIR		PN vs. STEP	
	Odds Ratio (95% CIs)	*p*	Odds Ratio (95% CIs)	*p*
Weaning off PN	1.1 (1.00–1.17)	0.03	1.13 (1.01, 1.31)	0.045
Survival	1.05 (1.01–1.09)	0.01	1.06 (0.99, 1.15)	0.051
Tx	1.14 (0.91, 1.44)	0.27	1.17 (0.86, 1.59)	0.31
LD	0.40 (0.11, 1.46)	0.21	1.19 (0.90, 1.58)	0.20
Model 1 *				
Weaning off PN	1.08 (1.01, 1.16)	0.03	1.12 (1.01,1.29)	0.048
Survival	1.15(0.9, 1.45)	0.11	1.64 (0.64, 2.39)	0.26
Tx	1.07 (0.80, 1.44)	0.51	1.01 (0.57, 1.9)	0.71
LD	1.24 (0.93, 1,63)	0.13	1.68 (0.61, 3.1)	0.28
Model 2 **				
Weaning off PN	1.18 (0.82, 1.13)	0.052	1.26 (0.97, 1.64)	0.07
Survival	1.04 (0.99, 1.11)	0.06	1.10 (0.98, 1.28)	0.09
Tx	0.78 (0.49, 1.21)	0.26	1.21 (0.41, 3.1)	0.78
LD	1.31 (0.95, 1.83)	0.10	1.23 (0.89,1.71)	0.39

PN: Parenteral nutrition; AGIR: autologous reconstructive gastro-intestinal surgery; STEP: serial transverse enteroplasty; CI: confidence interval; Tx: Transplantation; LD: liver disease; * Adjusted for age; ** Adjusted for age and primary diagnosis (gastroschisis).
